# Identifying miRNA sponge modules using biclustering and regulatory scores

**DOI:** 10.1186/s12859-017-1467-5

**Published:** 2017-03-14

**Authors:** Junpeng Zhang, Thuc D Le, Lin Liu, Jiuyong Li

**Affiliations:** 1 School of Engineering, Dali University, Dali, Yunnan 671003 People’s Republic of China; 2 School of Information Technology and Mathematical Sciences, University of South Australia, Mawson Lakes, SA 5095 Australia

**Keywords:** miRNA sponge, ceRNA, miRNA sponge module, Biclustering method, Regulatory score, Breast cancer

## Abstract

**Background:**

MicroRNA (miRNA) sponges with multiple tandem miRNA binding sequences can sequester miRNAs from their endogenous target mRNAs. Therefore, miRNA sponge acting as a decoy is extremely important for long-term loss-of-function studies both in vivo and *in silico*. Recently, a growing number of *in silico* methods have been used as an effective technique to generate hypotheses for in vivo methods for studying the biological functions and regulatory mechanisms of miRNA sponges. However, most existing *in silico* methods only focus on studying miRNA sponge interactions or networks in cancer, the module-level properties of miRNA sponges in cancer is still largely unknown.

**Results:**

We propose a novel *in silico* method, called miRSM (miRNA Sponge Module) to infer miRNA sponge modules in breast cancer. We apply miRSM to the breast invasive carcinoma (BRCA) dataset provided by The Cancer Genome Altas (TCGA), and make functional validation of the computational results. We discover that most miRNA sponge interactions are module-conserved across two modules, and a minority of miRNA sponge interactions are module-specific, existing only in a single module. Through functional annotation and differential expression analysis, we also find that the modules discovered using miRSM are functional miRNA sponge modules associated with BRCA. Moreover, the module-specific miRNA sponge interactions among miRNA sponge modules may be involved in the progression and development of BRCA. Our experimental results show that miRSM is comparable to the benchmark methods in recovering experimentally confirmed miRNA sponge interactions, and miRSM outperforms the benchmark methods in identifying interactions that are related to breast cancer.

**Conclusions:**

Altogether, the functional validation results demonstrate that miRSM is a promising method to identify miRNA sponge modules and interactions, and may provide new insights for understanding the roles of miRNA sponges in cancer progression and development.

**Electronic supplementary material:**

The online version of this article (doi:10.1186/s12859-017-1467-5) contains supplementary material, which is available to authorized users.

## Background

MicroRNAs (miRNAs) are small (~22 nt), single-stranded, non-coding RNA molecules which are involved in the post-transcriptional regulation of gene expression. By binding to target mRNAs, miRNAs typically cause degradation and translation repression of mRNAs [1]. The fine-tuning of gene regulation by miRNAs in a wide range of biological processes and tumor progressions has attracted significant attentions to understand the biological functions and regulatory mechanisms of miRNAs.

Recently, the competing endogenous effect at the post-transcriptional level has shifted our understanding of miRNA regulatory mechanism. There are several types of RNAs acting as competing endogenous RNAs (ceRNAs) (also called miRNA sponges or miRNA decoys) to prevent miRNAs from binding their authentic targets. These miRNA sponges include protein-coding RNAs, long non-coding RNAs (lncRNAs), pseudogenes and circular RNAs (circRNAs) [2–5]. More and more miRNA sponges in different biological conditions have been identified by biological experiments [6]. These miRNA sponges interact with each other via shared miRNAs, and the crosstalks between them are formed to develop miRNA-mediated interaction or miRNA sponge interaction network. However, similar to miRNA target prediction, the identification of miRNA sponge interaction networks by using biological experiments is limited by their low efficiency, time consumption and high cost. Thus, a growing number of computational methods have been proposed to identify miRNA sponge interaction networks.

Existing computational methods for identifying miRNA sponge interaction networks can be divided into three categories [7]: (1) pair-wise correlation approach, (2) partial association approach, and (3) mathematical modelling approach. In the first category [8–11], each pair of interacting miRNA sponges in a network have a significant positive correlation or there is a significant difference in their correlation between two different conditions. The main limitation of these methods is that they don’t consider the expression levels of the miRNAs shared by the two miRNA sponges when computing the correlation between the miRNA sponge pair. To address this limitation, methods of the second category [12–14] integrate the expression levels of the shared miRNAs of two miRNA sponges and calculate the partial association between them. These methods only use unweighted bipartite network consisting of putative miRNA-target interactions, but ignore the binding strengths between miRNAs and their targets. Moreover, some identified miRNA sponge interactions in the network are actually TF-target interactions or protein-protein interactions (PPIs), and should be removed. The third category [15–18] focuses on decribing a minimum or small miRNA sponge interaction network using different mathematical models. For each candidate miRNA sponge interaction, they would design a synthetic gene circuit to analyze the quantitative behavior of the miRNA sponge effect. The number of candidate miRNA sponge interactions is usually large. Since it is very time-consuming of designing many synthetic gene circuits for a large number of miRNA sponge interactions, these methods cannot be easily applied to study a larger miRNA sponge interaction network.

The identification of miRNA sponge interaction networks could provide a global view of studying the properties of miRNA sponges in cancer progression and development. Due to the modularity of cancer progression and development, it is also important to identify functional modules that involve miRNAs and miRNA sponges. Therefore, in this study, we present a novel computational method based on biclustering and regulatory scores to identify miRNA Sponge Modules (thus the proposed method is called miRSM).

We explore mRNA-related miRNA sponge modules by combining matched miRNA and mRNA expression data, and putative miRNA-target interactions. Instead of completely relying on putative miRNA-target interactions, we reconstruct miRNA-target interactions by considering both expression data and miRNA-target binding information. We use regulatory scores to infer miRNA-mRNA biclusters where a subset of mRNAs compete with each other to attract binding with a subset of miRNAs. We further identify miRNA sponge interactions in each miRNA-mRNA bicluster, and remove the candidate miRNA sponges which are not involved in any miRNA sponge interactions. The remaining candidate miRNA sponges and miRNAs in each bicluster are regarded as a miRNA sponge module.

The method is applied to the breast invasive carcinoma (BRCA) dataset provided by The Cancer Genome Altas (TCGA) to build miRNA sponge modules in BRCA. We discover that a few number of miRNA sponge interactions only exist in single module, and most miRNA sponge interactions are common across two modules. This result shows the module-conserved characteristic of miRNA sponge interactions across two different modules. Moreover, miRNA sponges of the modules are found to be biologically meaningful based on functional annotation and differential expression analysis. Through experimental validation using the thrid-party databases, some miRNA sponge interactions and miRNA-target interactions are experimentally validated. Finally, the comparison results show that miRSM performs better than or comparable to the other three existing methods (PC [8, 9], SPPC [12], and Hermes [13]) in identifying miRNA sponge interactions.

## Methods

### Data sources

The matched miRNA and mRNA expression data of human BRCA are obtained from Paci et al. [12]. The dataset is generated with the platform of TCGA level 3 IlluminaHiSeq in 72 matched tumor and normal tissues. miRNAs and mRNAs with missing values in >50% samples are removed from the dataset. The remaining missing values are imputed using the *k*-nearest neighbours (KNN) algorithm of the R package *impute*. Furthermore, we remove the mRNAs without gene symbols and take the average expression values of replicate miRNAs and mRNAs. Therefore, we obtain 453 miRNAs and 11,157 mRNAs in the 72 matched samples.

The putative miRNA-target interactions are from TargetScan v7.1 [19]. We retain those miRNA-target interactions with context++ scores less than 0 in TargetScan. The context++ score for each miRNA-target interaction is the sum of the contributions of 14 robustly selected features [19]. As a result, we obtain 228,423 interactions (with negative context++ scores) between 402 mature miRNAs and 12,441 mRNAs. In this study, we choose two representative databases of experimentally validated human TF-target interactions and PPIs to illustrate the method. The first database HTRIdb [20] is a popular repository of experimentally verified human transcriptional regulation interactions, and we collect 51,871 TF-target interactions from it. The second database HPRD v9 (Human Protein Reference Database) [21] is a well-cited human protein reference database with high-quality PPIs, and we obtain 36,852 protein-protein interactions (PPIs) from it.

A list of 40 Gene Ontology (GO) terms associated with 10 cancer hallmarks is from Plaisier et al. [22], and the gene sets of these hallmark-associated GO terms are obtained from MsigDB v5.1 [23]. The list of 2949 breast cancer genes are collected from COSMIC v77 [24], GAD [25], OMIM [26], BCGD [27] and G2SBC [28]. The list of 428 breast cancer miRNAs are obtained by integrating five databases: HMDD v2.0 [29], miR2Disease [30], miRCancer [31], oncomiRDB [32] and phenomiR v2.0 [33].

The experimentally validated miRNA-target interactions with strong evidence are from miRTarbase v6.1 [34]. The experimentally validated miRNA sponge interactions are retrived from [6, 7], and miRSponge [35], the first manually curated miRNA sponge interactions database. We only extract experimentally validated mRNA-related miRNA sponge interactions for validations.

### Overview of miRSM

**Fig. 1:**
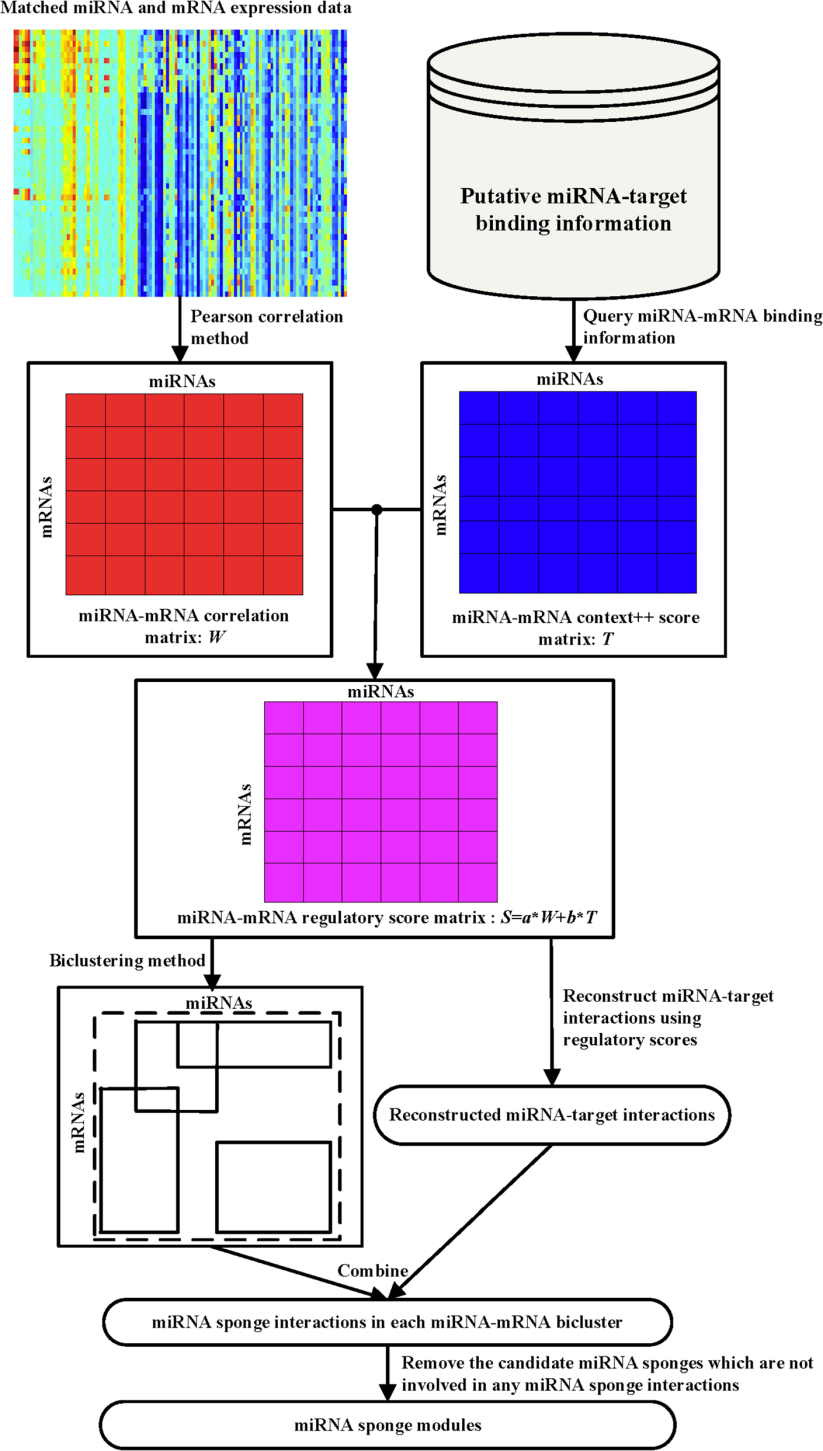
The pipeline of miRSM. We construct miRNA-mRNA correlation matrix using Pearson method, and miRNA-mRNA context++ score matrix using putative miRNA-target binding information. Next, miRNA-mRNA regulatory score matrix is inferred by combining miRNA-mRNA correlation and context++ score matrix. A biclustering method is then used to generate miRNA-mRNA biclusters. We identify miRNA sponge interactions in each miRNA-mRNA bicluster, and remove the candidate miRNA sponges which are not involved in any miRNA sponge interactions. The remaining candidate miRNA sponges and miRNAs in each bicluster are considered as a miRNA sponge module

Figure 1 depicts the pipeline of miRSM. The overall process of miRSM for identifying miRNA sponge modules includes the following steps:

1. Data preparation. We firstly collect expression profiles and miRNA-target binding information. The expression profiles of miRNAs and mRNAs, and the context++ scores of miRNA-target interactions are regarded as input dataset of the next step.
2. Create miRNA-mRNA regulatory score matrix. We firstly use Pearson correlation method to calculate miRNA-mRNA correlation matrix, *W*, of the matched miRNA and mRNA expression data. Based on the putative miRNA-target binding information retrieved from TargetScan, we generate the miRNA-mRNA context++ score matrix, *T*. By combining the correlation matrix and the context++ score matrix, we create miRNA-mRNA regulatory score matrix, *S*.
3. Infer miRNA-mRNA biclusters. The miRNA-mRNA regulatory score matrix is regarded as the input matrix of the biclustering method. A subset of mRNAs exhibit similar behavior across a subset of miRNAs in each bicluster.
4. Identify miRNA sponge modules. Pearson correlation method is used to compute the correlations of all possible mRNA-mRNA pairs of each bicluster. We use the regulatory scores between miRNAs and mRNAs to reconstruct miRNA-target interactions, and a hypergeometric test is utilized to evaluate the significance of the sharing of miRNAs by each mRNA-mRNA pair. The mRNA-mRNA pairs with significant sharing of miRNAs (*p*-value <0.01) and significant positive correlation (*p*-value <0.01) are regarded as candidate miRNA sponges and their interactions as candidate miRNA sponge interactions. Moreover, we remove the candidate miRNA sponge interactions that are actually TF-target interactions or PPIs and the candidate miRNA sponges which are not involved in any miRNA sponge interactions are removed too. Finally, the remaining candidate miRNA sponges and miRNAs in each bicluster are considered as a miRNA sponge module.

In the following, we will present the key steps in detail.

### Calculating miRNA-mRNA regulatory scores

The regulatory scores denote the degree of regulation between miRNAs and mRNAs considering both their correlations and context++ scores. The correlations between miRNAs and mRNAs are based on the matched miRNA and mRNA expression data, and the context++ scores of miRNA-mRNA interactions are extracted from TargetScan v7.1 [19]. Let *W* be miRNA-mRNA correlation matrix, and *T* be miRNA-mRNA context++ score matrix. The miRNA-mRNA regulatory score matrix *S* is calculated as follows:

1 $$ S=a*W+b*T $$

where *a* and *b* are tuning parameters with the value range of [0, 1], and the default values of them are set to 0.5, indicating that expression data and putative miRNA-target binding information contribute equally to the regulatory scores of miRNA-mRNA interactions. Since the value ranges of the elements of *W* and *T* are [−1, 1] and [−1, 0] respectively, when *a* and *b* take their default value (0.5), the (default) value range of the elements of *S* is [−1, 0.5].

In this study, we use the regulatory scores of miRNA-mRNA interactions to reconstruct putative miRNA-target interactions. We only consider negative values of the regulatory scores in *S* due to negative regulation of miRNAs. According to the empirical experiments, the negative correlation of two variables is around −0.3 under significant level of *p*-value <0.05. Thus, the default threshold *s* of regulatory scores is set to −0.3. That is to say, the miRNA-target interactions with regulatory scores equal to or less than *s* are regarded as reconstructed miRNA-target interactions.

### Identifying miRNA sponge modules

Given the miRNA-mRNA regulatory score matrix with *m* rows in *n* columns, the biclustering method allows simultaneous clustering the rows (mRNAs) and columns (miRNAs) of the matrix. Here, a bicluster corresponds a module. For each bicluster, a subset of mRNAs exhibit similar behavior across a subset of miRNAs. To identify miRNA-mRNA biclusters, a biclustering method called BCPlaid [36] is used. The BCPlaid is an improved version of Plaid model [37]. The Plaid model estimates the normal expression level of each gene, then infers biclusters of genes that have similarly unusual expression levels across the biclustered samples. This feature makes it an attractive method for clustering expression data. To improve the computationally efficient for fitting the Plaid model, the BCPlaid is presented based on speedy individual differences clustering and uses binary least squares to update the cluster membership parameters.

After obtaining the biclusters, we calculate correlations of all mRNA-mRNA pairs of each bicluster. For a given mRNA-mRNA pair (mR_1_ and mR_2_), the significance *p*-value of the shared miRNAs by these two mRNAs is calculated in the following.

2 $$ p=1-F\left(x\Big|N,M,K\right)=1-{\displaystyle \sum_{i=0}^{x-1}\frac{\left(\begin{array}{l}M\\ {}i\end{array}\right)\left(\begin{array}{l}N-M\\ {}K-i\end{array}\right)}{\left(\begin{array}{l}N\\ {}K\end{array}\right)}} $$

In the formula, *N* is the number of all miRNAs in the dataset, *M* and *K* represent the total numbers of miRNAs regulating mR_1_ and mR_2_ respectively, and *x* is the number of common miRNAs shared by mR_1_ and mR_2_. The mRNA-mRNA pairs with significant sharing of miRNAs (*p*-value <0.01) and significant positive correlations (*p*-value <0.01) are regarded as candidate miRNA sponge interactions. We further remove the candidate miRNA sponge interactions that are actually TF-target interactions or PPIs and the candidate miRNA sponges which are not involved in any miRNA sponge interactions are removed too. Finally, all the reserved miRNA sponges and miRNAs in a bicluster are regarded as a miRNA sponge module.

## Results and Discussion

### miRNA sponge modules for BRCA

**Fig. 2:**
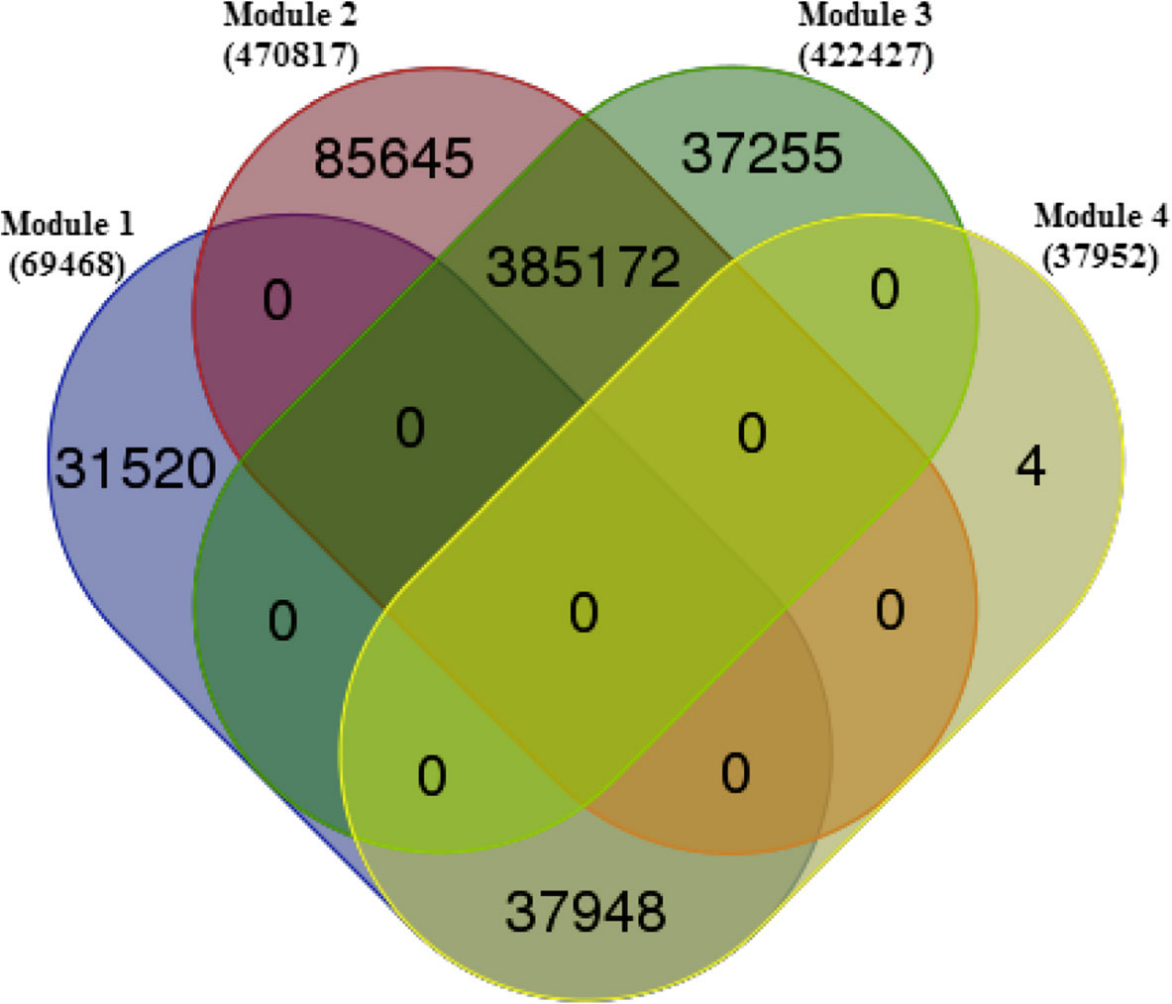
Overlaps and differences of miRNA sponge interactions in the four miRNA sponge modules

The default values of the tuning parameters *a* and *b* are set to 0.5, and the threshold *s* of regulatory scores is set to −0.3 for the reconstruction of miRNA-target interactions. As shown in Table 1, we identify four miRNA sponge modules. As illustrated in Fig. 2, there are many common miRNA sponge interactions (385,172) between Module 2 and Module 3, and almost all miRNA sponge interactions (37,948) in Module 4 exist in Module 1. However, there is no overlap of miRNA sponge interactions among the four modules. This result implies that most miRNA sponge interactions tend to be module-conserved across two modules, and a small portion of miRNA sponge interactions are module-specific (i.e. only exist in a single module). The detail information of the four modules and module-specific miRNA sponge interactions can be seen in Additional file 1.

**Table 1:** miRNA sponge modules in BRCA

miRSM	#miRNAs	#miRNA sponges	#miRNA sponge interactions
Module 1	110	546	69468
Module 2	130	1213	470817
Module 3	92	1142	422427
Module 4	105	354	37952

### miRNA sponge modules are biologically meaningful

As described previously (see the Data sources section), we collect a list of 428 BRCA miRNAs and 2949 BRCA genes. We also collect a list of 40 unique GO terms associated with 10 cancer hallmarks (Self Sufficiency in Growth Signals, Insensitivity to Antigrowth Signals, Evading Apoptosis, Limitless Replicative Potential, Sustained Angiogenesis, Tissue Invasion and Metastasis, Genome Instability and Mutation, Tumor Promoting Inflammation, Reprogramming Energy Metabolism, and Evading Immune Detection). Only five cancer hallmarks (Self Sufficiency in Growth Signals, Insensitivity to Antigrowth Signals, Evading Apoptosis, Tissue Invasion and Metastasis, and Genome Instability and Mutation) have related gene sets in more than half of the associated GO terms (details in Additional file 2). As a result, we have a list of 2224 unique genes associated with the five representative cancer hallmarks. The list of BRCA miRNAs, BRCA genes, and cancer hallmark genes can be seen in Additional file 3.

**Fig. 3:**
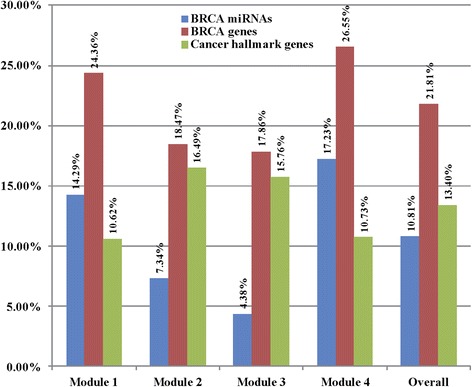
The percentage of BRCA miRNAs, BRCA miRNA sponges, and cancer hallmark miRNA sponges in the four miRNA sponge modules

As shown in Fig. 3, the percentages of BRCA miRNAs, BRCA genes, and cancer hallmark genes are different due to the different components of each miRNA sponge module. Overall, 10.81% of miRNAs are BRCA miRNAs, 21.81% of miRNA sponges are BRCA genes, and 13.40% of miRNA sponges are cancer hallmark genes in the identified miRNA sponge modules.

Since differentially expressed genes with abnormal expression are closely associated with the occurrence and development of cancer, we also perform differential expression analysis on the BRCA expression profiles using *limma* package [38] of Bioconductor. As a result, 278 miRNAs (adjusted *p*-value <0.01, adjusted by Benjamini & Hochberg method), and 5602 mRNAs (adjusted *p*-value <1E-04) are identified to be differentially expressed at significant level (details in Additional file 4). We find that the miRNA sponges in the four miRNA sponge modules are all differentially expressed mRNAs, and the percentages of differentially expressed miRNAs of Module 1 to Module 4 are 54.55% (60 out of 110), 41.54% (54 out of 130), 61.96% (57 out of 92) and 60.95% (64 out of 105), respectively. This result indicates that the identified modules are functional miRNA sponge modules, and may be closely associated with the occurrence and development of BRCA.

To uncover the biological machanism in BRCA, we further conduct functional annotation analysis of the miRNA sponges using GeneCodis [39] (the online tool at http://genecodis.cnb.csic.es/). The top 5 enriched GO (Gene Ontology) [40] terms and KEGG (Kyoto Encyclopedia of Genes and Genomes) [41] pathways are listed in Table 2.

**Table 2:** Top 5 enriched GO terms and KEGG pathways for the miRNA sponges in each module and module-specific interactions

miRSM	Items	#miRNA sponges	Adjusted *p*-value
Module 1	GO:0000278-Mitotic cell cycle	45	7.36E-28
	GO:0051301-Cell division	43	4.10E-27
	GO:0007049-Cell cycle	41	4.61E-18
	GO:0007067-Mitosis	28	2.50E-17
	GO:0000236-Mitotic prometaphas	20	3.59E-16
	KEGG:04110-Cell cycle	17	7.73E-10
	KEGG:04914-Progesterone-mediated oocyte maturation	9	0.000432
	KEGG:05110-Vibrio cholerae infection	7	0.000676
	KEGG:03060-Protein export	5	0.000728
	KEGG:04114-Oocyte meiosis	9	0.001271
Module 2	GO:0007165-Signal transduction	124	2.37E-26
	GO:0007275-Multicellular organismal development	106	1.41E-24
	GO:0045944-Positive regulation of transcription from RNA polymerase II promoter	73	2.16E-19
	GO:0000122-Negative regulation of transcription from RNA polymerase II promoter	55	3.39E-15
	GO:0007155-Cell adhesion	62	9.26E-14
	KEGG:04060-Cytokine-cytokine receptor interaction	35	5.45E-10
	KEGG:04080-Neuroactive ligand-receptor interaction	33	1.40E-08
	KEGG:05200-Pathways in cancer	36	2.53E-08
	KEGG:04510-Focal adhesion	26	1.40E-07
	KEGG:04144-Endocytosis	25	3.30E-07
Module 3	GO:0007165-Signal transduction	113	1.01E-22
	GO:0045944-Positive regulation of transcription from RNA polymerase II promoter	73	6.85E-21
	GO:0007275-Multicellular organismal development	94	5.77E-20
	GO:0000122-Negative regulation of transcription from RNA polymerase II promoter	52	1.98E-14
	GO:0007155-Cell adhesion	58	9.76E-13
	KEGG:04060-Cytokine-cytokine receptor interaction	31	4.46E-08
	KEGG:04080-Neuroactive ligand-receptor interaction	31	5.24E-08
	KEGG:05200-Pathways in cancer	34	6.92E-08
	KEGG:04920-Adipocytokine signaling pathway	15	9.78E-08
	KEGG:04010-MAPK signaling pathway	29	2.07E-07
Module 4	GO:0000278-Mitotic cell cycle	42	2.03E-32
	GO:0051301-Cell division	40	2.79E-31
	GO:0000236-Mitotic prometaphase	20	6.71E-20
	GO:0007067-Mitosis	26	7.54E-20
	GO:0006260-DNA replication	24	8.54E-20
	KEGG:04110-Cell cycle	14	2.48E-09
	KEGG:03060-Protein export	5	0.00015
	KEGG:05110-Vibrio cholerae infection	6	0.000517
	KEGG:04914-Progesterone-mediated oocyte maturation	6	0.003029
	KEGG:04141-Protein processing in endoplasmic reticulum	8	0.003089
Module-specific	GO:0007275-Multicellular organismal development	146	6.67E-30
	GO:0007165-Signal transduction	165	2.46E-29
	GO:0045944-Positive regulation of transcription from RNA polymerase II promoter	95	5.49E-21
	GO:0000122-Negative regulation of transcription from RNA polymerase II promoter	72	7.02E-17
	GO:0007264-Small GTPase mediated signal transduction	58	6.02E-15
	KEGG:05200-Pathways in cancer	56	2.26E-13
	KEGG:04510-Focal adhesion	39	2.92E-11
	KEGG:04144-Endocytosis	34	1.77E-08
	KEGG:04060-Cytokine-cytokine receptor interaction	39	7.93E-08
	KEGG:04810-Regulation of actin cytoskeleton	34	9.26E-08

The *p*-values are adjusted by Benjamini-Hochberg (BH) method

As shown in Table 2, most enriched GO biological processes and KEGG pathways are shared by Module 2 and Module 3, and there also exist common pathways between Module 1 and Module 4. This suggests that similar modules (Module 2 and Module 3, Module 1 and Module 4) with many overlaps of miRNA sponge interactions tend to have similar biological functions, and vice versa. Moreover, all modules have many enriched GO biological processes and KEGG pathways related to BRCA, such as Signal transduction (GO:0007165) [42], Cell cycle (GO:0007049, KEGG:04110) [43], and Pathways in cancer (KEGG:05200). Since the BRCA dataset is a cancer dataset, the result demonstrates that the discovered miRNA sponge modules are closely associated with the biological condition of the dataset. The module-specific miRNA sponge interactions among four modules are also significantly enriched in Signal transduction (GO:0007165) and Pathways in cancer (KEGG:05200). The result indicates that these module-specific miRNA sponge interactions may be involved in the progression and development of BRCA.

In summary, miRNA sponge modules are biologically significant, which may imply that the miRNA sponge modules discovered based on the BRCA dataset can indeed reveal the biological mechanism in BRCA. The detailed information of significant GO terms and KEGG pathways for the miRNA sponges in each module and module-specific interactions can be found in Additional file 5.

### Validation of the interactions in the miRNA sponge modules

In this section, we validate two types of interactions (miRNA sponge interactions and miRNA-target interactions) in the identified miRNA sponge modules. For the ground truth of validation, we have collected 46 experimentally validated mRNA-related miRNA sponge interactions, and 5195 experimentally validated miRNA-target interactions with strong evidence (details in Additional file 6). For the validation of miRNA sponge interactions, Module 2 has five experimentally validated miRNA sponge interactions from a small number (46) of ground truth interactions. They are all PTEN-related miRNA sponge interactions (five genes including KLF6, LRCH1, MBNL1, SERINC1 and ZEB2 compete with PTEN). In the case of the validation of miRNA-target interactions, the numbers of experimentally validated miRNA-target interactions with strong evidence in Module 1 and Module 3 achieve 17 and 71, respectively. The detailed information can be seen in Additional file 7.

### Comparison with other existing methods in identifying miRNA sponge interactions

In this section, we compare the performance of miRSM with other existing methods in terms of the numbers of breast cancer-related miRNA sponge interactions and experimentally validated miRNA sponge interactions in the findings by the methods. We define that breast cancer-related miRNA sponge interactions are those in which the two interactive parties exist in the list of 2949 breast cancer genes (i.e. the breast cancer genes collected in the Data sources section). Since the mathematical modelling approaches in the third category are only applied to study a small number of miRNA sponge interactions, we don’t compare miRSM with them in this study. Therefore, in this study, we select three typical methods from the first two categories (pair-wise correlation approach and partial association approach) for the comparison study. The first method is the Positive Correlation (PC) method [8, 9], which is based on the positive correlation between each pair of interacting miRNA sponges. The second method is the Sensitivity Partial Pearson Correlation (SPPC) method [12], which uses partial correlations to estimate the contributed effect of common miRNAs on miRNA sponge interacting pairs. The third method is Hermes [13], which uses conditional mutual information to estimate partial associations between miRNA sponges.

To make a fair comparison, we use the same *p*-value cutoff (0.01) to calculate significance of the findings of shared miRNAs and the positive correlations of possible miRNA sponge interaction pairs. For the SPPC method, the cutoff of *sensitivity correlation* (the difference between Pearson Correlation and Partial Pearson Correlation) is set to 0.3, which is the value used in [12].

We compare the results of miRSM with three different parameter settings with those of the other 3 methods. As shown in Table 3, the numbers of validated miRNA sponge interactions for the three different parameter settings of miRSM are all 5, indicating a stable validation results of our method. In the case of the number of validated miRNA sponge interactions, our method performs better than SPPC and Hermes, but slightly worse than PC. However, our method generally performs better than the other three methods in the percentage of breast cancer-related miRNA sponge interactions.

**Table 3:** Comparison with other existing three methods in the number of breast cancer-related miRNA sponge interactions and experimentally validated miRNA sponge interactions

Methods	#Interactions	#Breast cancer-related interactions (percentage)	Validated miRNA sponge interactions	#Validated interactions
miRSM_default	577544	21669 (3.75%)	SERINC1:PTEN, LRCH1:PTEN, KLF6:PTEN, ZEB2:PTEN, MBNL1:PTEN	5
miRSM_v1	169617	6104 (3.60%)	SERINC1:PTEN, LRCH1:PTEN, KLF6:PTEN, ZEB2:PTEN, MBNL1:PTEN	5
miRSM_v2	1228533	46186 (3.76%)	SERINC1:PTEN, LRCH1:PTEN, KLF6:PTEN, ZEB2:PTEN, MBNL1:PTEN	5
PC	933516	28354 (3.04%)	HIAT1:PTEN, SERINC1:PTEN, KLF6:PTEN, TNKS2: PTEN, PDGFRA:RB1, LRCH1:PTEN, AFF1:PTEN	7
SPPC	177371	6434 (3.63%)	LRCH1:PTEN, KLF6:PTEN	2
Hermes	43144	1018 (2.36%)	JARID2:PTEN, RUNX1:PTEN, AFF1:PTEN	3

“:” denotes “competing with”. Three miRSM networks including miRSM_default with *a* = *b* = 0.5 and *s* = -0.3, miRSM_v1 with *a* = 0.45, *b* = 0.55 and *s* = -0.3, and miRSM_v2 with *a* = 0.55, *b* = 0.45 and *s* = -0.3 are used to compare

**Fig. 4:**
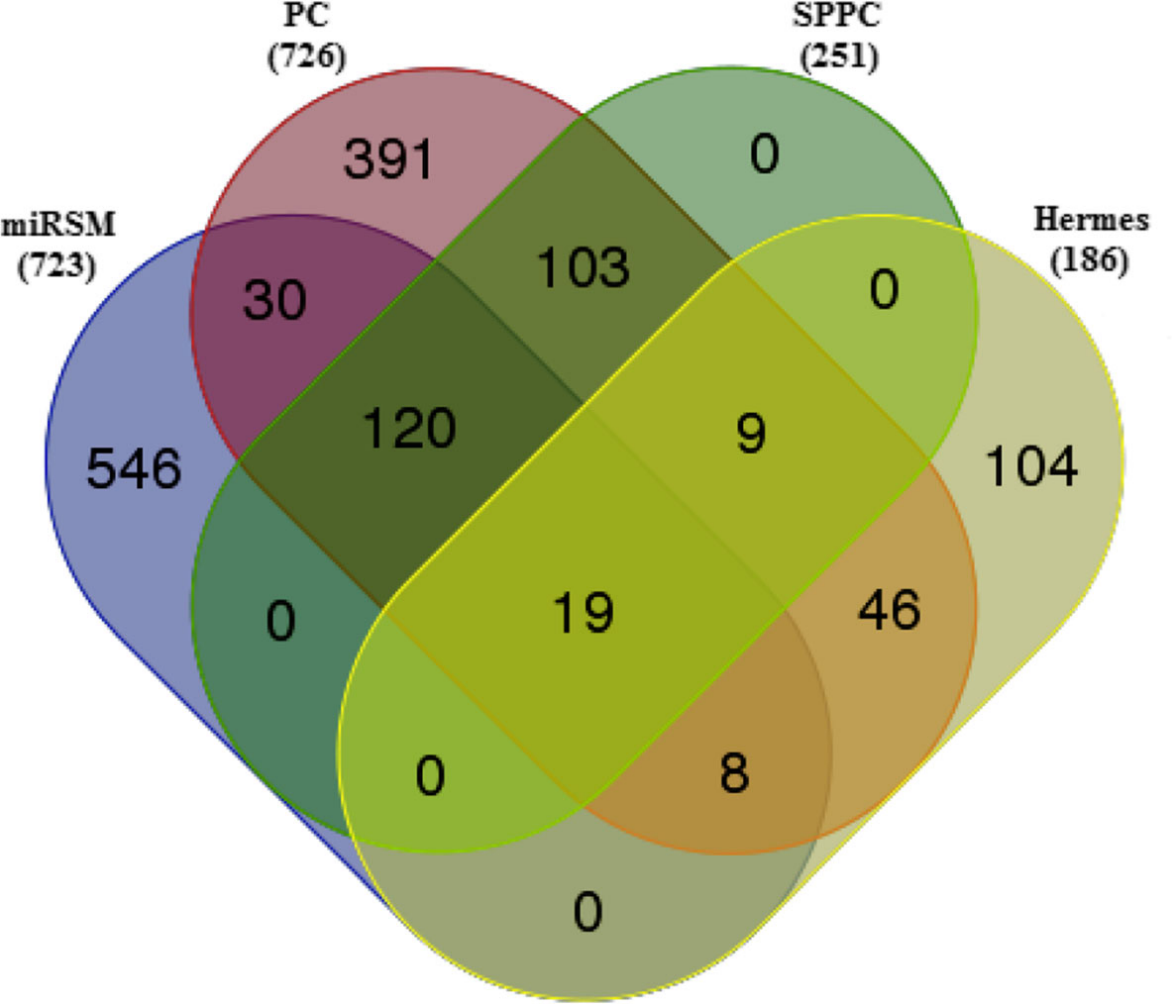
Overlaps and differences between PTEN-related miRNA sponge interactions identified by miRSM, PC, SPPC, and Hermes

Since PTEN-related miRNA sponge interactions are widely studied, we further focus on studying the overlap and differences between PTEN-related miRNA sponge interactions identified by miRSM_default, PC, SPPC, and Hermes. Figure 4 illustrates that different computational methods identify different sets of PTEN-related miRNA sponge interactions. Specifically, many PTEN-related miRNA sponge interactions are only inferred by miRSM.

## Conclusions

miRNA sponge effect is a novel type of gene regulation at the post-transcriptional level. The crosstalks between miRNA sponges involve many classes of RNAs, mainly including protein-coding and non-coding RNAs. Among different types of miRNA sponges (protein-coding RNAs, lncRNAs, pseudogenes, circRNAs, etc), the vast majority of them are protein-coding RNAs. Thus, we focus on mRNA-related miRNA sponge modules in this study.

Identifying miRNA sponge interaction network using *in silico* methods is an emerging research field. The fundamental principle of the identification of miRNA sponge interactions using *in silico* methods are based on experimental evidence for miRNA sponges. The basic experimental evidence for miRNA sponges is that the overexpression of the putative miRNA sponges leads to increased expression of the competing RNAs, and vice versa. That is to say, miRNA sponge interaction pairs are positively correlated at expression level. Until now, an ubiquitous limitation of *in silico* methods assessing miRNA sponge interactions is that they are wholly dependent upon unweighted miRNA-target interactions at sequence level, and rarely take expression level into account. In fact, integrating both sequence level and expression level information lead to the discovery of more candidate miRNA sponge interaction pairs when exploring miRNA sponge interaction networks. In addition, an underlying problem of existing *in silico* methods is that they also regard other known gene regulatory interactions or molecular interactions (e.g. TF-target interactions and PPIs) as miRNA sponge interactions. Actually, these interactions are direct interactions rather than crosstalks between miRNA sponges.

miRNA sponge interaction networks provide a global way to study the biological functions of miRNA sponges in cancer. Since modularity is an important feature of cancer progression and development, it is extremly necessary to investigate functional miRNA sponge modules associated with cancer from a local point of view. Therefore, in this paper, we propose miRSM to identify miRNA sponge modules. The method integrates data source from both sequence level and expression level, and uses regulatory scores to reconstruct miRNA-target interactions and infer miRNA-mRNA biclusters in which a subset of mRNAs compete with each other to bind with a subset of miRNAs. Moreover, we remove miRNA sponge interactions that are experimentally validated TF-target interactions or PPIs to improve the prediction of miRNA sponge modules.

miRSM is a parametric method, i.e. the identified miRSM modules and the validation results are closely related with the tuning parameters *a* and *b*, and the threshold *s* of regulatory scores. As shown in Table 4, the threshold *s* is a negative value, and is associated with the number of candidate miRNA sponges. The smaller the value of *s* is, the less the number of miRNA sponge interactions is. The parameters *a* and *b* denote the contributions of expression data and sequence data to the identification of miRNA-target interactions, and the default values of *a* and *b* are the same. If *a* > *b*, the number of miRNA sponge interactions will increase, and vice versa.

**Table 4:** The number of identified miRNA sponge interactions and experimentally validated miRNA sponge interactions under different parameter settings

Parameter settings of miRSM	#Interactions	Validated miRNA sponge interactions	#Validated interactions
*a* = 0.5, *b* = 0.5, *s* = -0.3	577544	SERINC1:PTEN, LRCH1:PTEN, KLF6:PTEN, ZEB2:PTEN, MBNL1:PTEN	5
*a* = 0.5, *b* = 0.5, *s* = -0.25	2033124	AFF1:PTEN, ZEB2:PTEN, SERINC1:PTEN, MBNL1:PTEN, LRCH1:PTEN, KLF6:PTEN, FN1: VCAN	7
*a* = 0.5, *b* = 0.5, *s* = -0.35	61161	/	0
*a* = 0.5, *b* = 0.5, *s* = -0.4	1041	/	0
*a* = 0.5, *b* = 0.5, *s* = -0.45	84	/	0
*a* = 0.4, *b* = 0.6, *s* = -0.3	17972	LRCH1:PTEN, KLF6:PTEN	2
*a* = 0.45, *b* = 0.55, *s* = -0.3	169617	SERINC1:PTEN, LRCH1:PTEN, KLF6:PTEN, ZEB2:PTEN, MBNL1:PTEN	5
*a* = 0.55, *b* = 0.45, *s* = -0.3	1228533	SERINC1:PTEN, LRCH1:PTEN, KLF6:PTEN, ZEB2:PTEN, MBNL1:PTEN	5
*a* = 0.6, *b* = 0.4, *s* = -0.3	2029912	AFF1:PTEN, ZEB2:PTEN, SERINC1:PTEN, MBNL1:PTEN, LRCH1:PTEN, KLF6:PTEN, FN1:VCAN	7

The comparison results show that miRSM performs better than or comparable to the other three existing methods (PC, SPPC, Hermes). Different methods have their own merits, leading to different sets of miRNA sponge interactions. The results focusing on PTEN-related miRNA sponge interactions show that miRSM can identify many different miRNA sponge interactions from the other three methods.

In summary, miRSM can be a promising method for identifying miRNA sponge modules, and hence provides new insights into the regulatory mechanisms and functions of miRNA sponges in different biological processes, including pathogenesis of cancers.

## Additional files

Additional file 1: miRNA sponge modules and module-specific miRNA sponge interactions. Four modules are identified by miRSM with the default parameter setting. The module-specific miRNA sponge interactions only exist in a single miRNA sponge module. (XLSX 15 mb)

Additional file 2: GO terms and related genes associated with 10 hallmarks of cancer. There are 40 unique GO terms associated with 10 hallmarks of cancer. Only 5 cancer hallmarks (Self Sufficiency in Growth Signals, Insensitivity to Antigrowth Signals, Evading Apoptosis, Tissue Invasion and Metastasis, and Genome Instability and Mutation) have related gene sets in more than half associated GO terms. (XLSX 72 kb)

Additional file 3: The list of BRCA miRNAs, BRCA genes, and cancer hallmark genes. There are 428 BRCA miRNAs, 2949 BRCA genes and 2224 cancer hallmark genes. (XLSX 77 kb)

Additional file 4: Differentially expressed miRNAs and mRNAs in BRCA dataset. The *p*-values are adjusted by Benjamini-Hochberg (BH) method. We identify 278 miRNAs (adjusted *p*-value <0.01), and 5602 mRNAs (adjusted *p*-value <1E-04) to be differentially expressed at significant level. (XLSX 1 mb)

Additional file 5: The significant GO terms and KEGG pathways for miRNA sponges in each module and module-specific interactions. The *p*-values are adjusted by Benjamini-Hochberg (BH) method, and the *p*-value cutoff is set to 0.05. (XLSX 209 kb)

Additional file 6: Experimentally validated mRNA-related miRNA sponge interactions and miRNA-target interactions with strong evidence. After removing replicate interactions, we have collected 46 experimentally validated mRNA-related miRNA sponge interactions, and 5195 experimentally validated miRNA-target interactions with strong evidence for validation. (XLSX 152 kb)

Additional file 7: Experimentally validated miRNA sponge interactions and miRNA-target interactions in the modules identified by miRSM. (XLSX 11 kb)

## Abbreviations

BH: Benjamini-hochberg
BRCA: Breast invasive carcinoma
ceRNA: Competing endogenous RNA
circRNA: Circular RNA
GO: Gene ontology
HPRD: Human protein reference database
KEGG: Kyoto encyclopedia of genes and genomes
KNN: *k*-nearest neighbours
lncRNA: Long non-coding RNA
miRNA: microRNA
miRSM: miRNA sponge module
PC: Positive correlation
PPI: Protein-protein interaction
SPPC: Sensitivity partial pearson correlation
TCGA: The cancer genome altas

## References

[1] Bartel DP (2009). MicroRNAs: target recognition and regulatory functions. Cell.

[2] Poliseno L, Salmena L, Zhang J (2010). A coding-independent function of gene and pseudogene mRNAs regulates tumour biology. Nature.

[3] Cesana M, Cacchiarelli D, Legnini I (2011). A long noncoding RNA controls muscle differentiation by functioning as a competing endogenous RNA. Cell.

[4] Hansen TB, Jensen TI, Clausen BH (2013). Natural RNA circles function as efficient microRNA sponges. Nature.

[5] Memczak S, Jens M, Elefsinioti A (2013). Circular RNAs are a large class of animal RNAs with regulatory potency. Nature.

[6] Tay Y, Rinn J, Pandolfi PP (2014). The multilayered complexity of ceRNA crosstalk and competition. Nature.

[7] Le TD, Zhang J, Liu L, Li J. Computational methods for identifying miRNA sponge interactions. Briefings Bioinf. 2016, doi: 10.1093/bib/bbw042. 10.1093/bib/bbw042 27273287

[8] Zhou X, Liu J, Wang W (2014). Construction and investigation of breast-cancer-specific ceRNA network based on the mRNA and miRNA expression data. IET Syst Biol.

[9] Xu J, Li Y, Lu J (2015). The mRNA related ceRNA-ceRNA landscape and significance across 20 major cancer types. Nucleic Acids Res.

[10] Shao T, Wu A, Chen J (2015). Identification of module biomarkers from the dysregulated ceRNA-ceRNA interaction network in lung adenocarcinoma. Mol Biosyst.

[11] Chiu YC, Hsiao TH, Chen Y (2015). Parameter optimization for constructing competing endogenous RNA regulatory network in glioblastoma multiforme and other cancers. BMC Genomics.

[12] Paci P, Colombo T, Farina L (2014). Computational analysis identifies a sponge interaction network between long non-coding RNAs and messenger RNAs in human breast cancer. BMC Syst Biol.

[13] Sumazin P, Yang X, Chiu HS (2011). An extensive microRNA-mediated network of RNA-RNA interactions regulates established oncogenic pathways in glioblastoma. Cell.

[14] Chiu HS, Llobet-Navas D, Yang X (2015). Cupid: simultaneous reconstruction of microRNA-target and ceRNA networks. Genome Res.

[15] Figliuzzi M, Marinari E, De Martino A (2013). MicroRNAs as a selective channel of communication between competing RNAs: a steady-state theory. Biophys J.

[16] Bosia C, Pagnani A, Zecchina R (2013). Modelling competing endogenous RNA networks. PLoS One.

[17] Ala U, Karreth FA, Bosia C (2013). Integrated transcriptional and competitive endogenous RNA networks are cross-regulated in permissive molecular environments. Proc Natl Acad Sci U S A.

[18] Yuan Y, Liu B, Xie P (2015). Model-guided quantitative analysis of microRNA-mediated regulation on competing endogenous RNAs using a synthetic gene circuit. Proc Natl Acad Sci U S A.

[19] Agarwal V, Bell GW, Nam JW (2015). Predicting effective microRNA target sites in mammalian mRNAs. Elife.

[20] Bovolenta LA, Acencio ML, Lemke N (2012). HTRIdb: an open-access database for experimentally verified human transcriptional regulation interactions. BMC Genomics.

[21] Keshava Prasad TS, Goel R, Kandasamy K (2009). Human protein reference database--2009 update. Nucleic Acids Res.

[22] Plaisier CL, Pan M, Baliga NS (2012). A miRNA-regulatory network explains how dysregulated miRNAs perturb oncogenic processes across diverse cancers. Genome Res.

[23] Subramanian A, Tamayo P, Mootha VK (2005). Gene set enrichment analysis: a knowledge-based approach for interpreting genome-wide expression profiles. Proc Natl Acad Sci U S A.

[24] Futreal PA, Coin L, Marshall M (2004). A census of human cancer genes. Nat Rev Cancer.

[25] Becker KG, Barnes KC, Bright TJ (2004). The genetic association database. Nat Genet.

[26] Hamosh A, Scott AF, Amberger JS (2005). Online mendelian inheritance in Man (OMIM), a knowledgebase of human genes and genetic disorders. Nucleic Acids Res.

[27] Baasiri RA, Glasser SR, Steffen DL (1999). The breast cancer gene database: a collaborative information resource. Oncogene.

[28] Mosca E, Alfieri R, Merelli I (2010). A multilevel data integration resource for breast cancer study. BMC Syst Biol.

[29] Lu M, Zhang Q, Deng M (2008). An analysis of human microRNA and disease associations. PLoS One.

[30] Jiang Q, Wang Y, Hao Y (2009). miR2Disease: a manually curated database for microRNA deregulation in human disease. Nucleic Acids Res.

[31] Xie B, Ding Q, Han H (2013). miRCancer: a microRNA-cancer association database constructed by text mining on literature. Bioinformatics.

[32] Wang D, Gu J, Wang T (2014). OncomiRDB: a database for the experimentally verified oncogenic and tumor-suppressive microRNAs. Bioinformatics.

[33] Ruepp A, Kowarsch A, Schmidl D (2010). PhenomiR: a knowledgebase for microRNA expression in diseases and biological processes. Genome Biol.

[34] Chou CH, Chang NW, Shrestha S (2016). miRTarBase 2016: updates to the experimentally validated miRNA-target interactions database. Nucleic Acids Res.

[35] Wang P, Zhi H, Zhang Y, et al. miRSponge: a manually curated database for experimentally supported miRNA sponges and ceRNAs. Database: the journal of biological databases and curation. 2015; doi: 10.1093/database/bav098. 10.1093/database/bav098 PMC4589693 26424084

[36] Turner H, Bailey T, Krzanowski W (2005). Improved biclustering of microarray data demonstrated through systematic performance tests. Comput Stat Data Anal.

[37] Lazzeroni L, Owen A (2002). Plaid models for gene expression data. Stat Sin.

[38] Ritchie ME, Phipson B, Wu D (2015). Limma powers differential expression analyses for RNA-sequencing and microarray studies. Nucleic Acids Res.

[39] Tabas-Madrid D, Nogales-Cadenas R, Pascual-Montano A (2012). GeneCodis3: a non-redundant and modular enrichment analysis tool for functional genomics. Nucleic Acids Res.

[40] Ashburner M, Ball CA, Blake JA (2000). Gene ontology: tool for the unification of biology. Nat Genet.

[41] Kanehisa M, Goto S (2000). KEGG: kyoto encyclopedia of genes and genomes. Nucleic Acids Res.

[42] Krauss G. Biochemistry of Signal Transduction and Regulation. 4th ed. Hoboken: Wiley-VCH; 2008.

[43] Yu Z, Baserga R, Chen L (2010). microRNA, cell cycle, and human breast cancer. Am J Pathol.

